# Number of sons contributes to ageing-associated inflammation

**DOI:** 10.1038/srep08631

**Published:** 2015-02-27

**Authors:** Saara Marttila, Tapio Nevalainen, Laura Kananen, Juulia Jylhävä, Marja Jylhä, Antti Hervonen, Jorma Ilonen, Mikko Hurme

**Affiliations:** 1 Department of Microbiology and Immunology, School of Medicine, University of Tampere, Tampere, Finland; 2 Gerontology Research Center, Tampere, Finland; 3 School of Health Sciences, University of Tampere, Tampere, Finland; 4 Immunogenetics Laboratory, University of Turku, Turku, Finland; 5 Department of Clinical Microbiology, University of Eastern Finland, Kuopio, Finland; 6 Fimlab Laboratories, Tampere, Finland

## Abstract

The rate of inflammation increases in elderly individuals, a phenomenon called inflammaging, and is associated with degenerative diseases. However, the causes of inflammaging and the origin of the associated inflammatory mediators have remained enigmatic. We show herein that there is a positive correlation between the number of sons born and C-reactive protein concentrations in 90-year-old women. This association is influenced by HLA genetics known to regulate the immune response against HY antigens.

Ageing is associated with a gradual degradation of immune defense, affecting both innate and adaptive mechanisms^1^. One of the most striking ageing-associated changes is the increase in the level of inflammatory mediators, called inflammaging^2^,^3^, which manifests as an increase in the blood levels of several, most likely all, markers of inflammation. However, the basic mechanisms of this process are still poorly understood. Although inflammaging is associated with several degenerative diseases or ageing phenotypes, the causality is not clear, i.e., it is unclear whether the inflammation is induced by degenerative processes or whether it has a causative role in pathogenesis. Moreover, the cell types producing these elevated mediator levels are not known, though it is known that blood monocyte/macrophages from aged individuals often produce lower amounts of inflammatory mediators than cells from younger individuals^3^.

It is probable that several life-style, environmental and nutritional factors as well as long-lasting exposure to microbes have an influence on the development of inflammaging^3^. We hypothesised that the number of children born, a common factor modifying the biology of women, would be connected to inflammaging. This hypothesis is based on several previous findings. First, there are several reports demonstrating the adverse effects of parity on the health of women, suggesting that there is a trade-off between the energy invested in somatic maintenance and in reproduction, though the data are not consistent^4^,^5^. Some data also suggest that the sex of the child may have an influence on maternal longevity. Indeed, there is epidemiological evidence showing that the number of sons, but not daughters, is associated with a shortened life-span, which was first described in a historic 17th–18th century Sami population in northern Scandinavia^6^,^7^. In addition to this, it is also known that cells derived from the male fetus can induce a classical T cell response in the mother against the Y chromosomal transplantation antigen (HY) and that this response may be associated with complications in ensuing pregnancies^8^,^9^.

## Results

**Figure 1:**
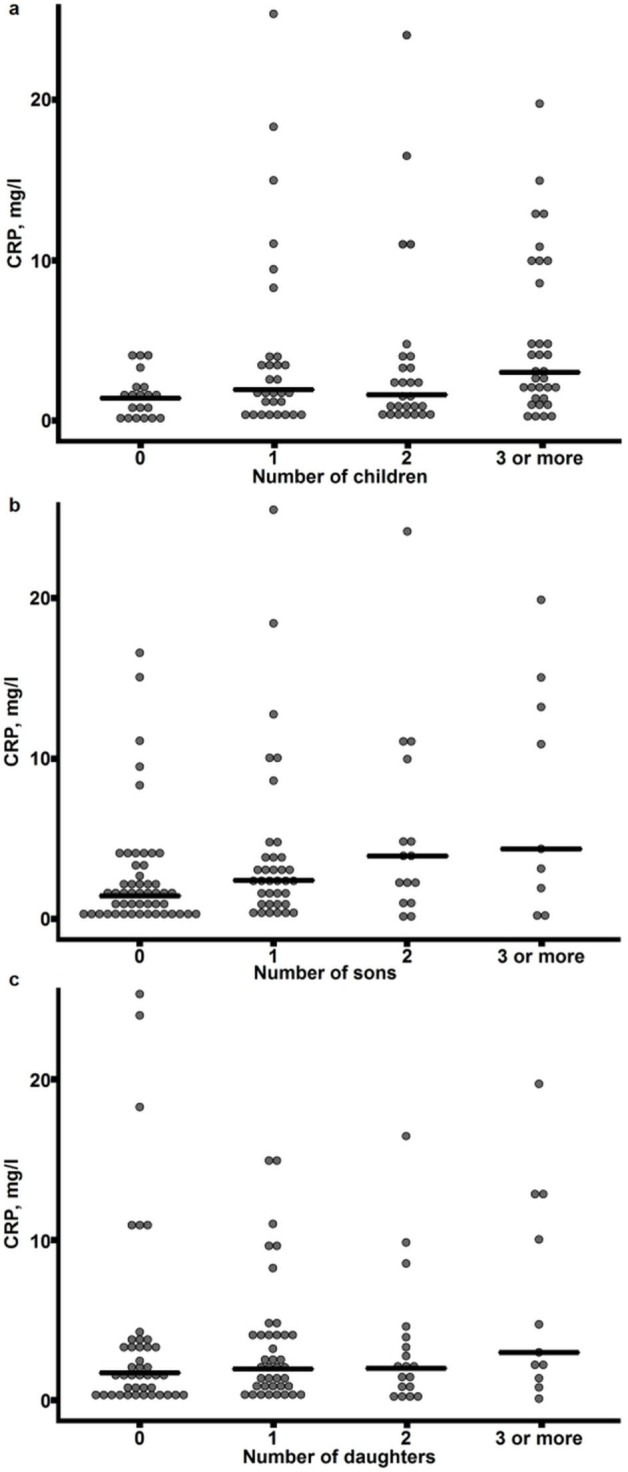
CRP concentration and number of progeny. CRP concentration in nonagenarian women grouped according to the number of children (a), sons (b) and daughters (c). Each dot represents one individual, and the horizontal line corresponds to the median of each group.

To test this hypothesis, we quantified inflammaging in a cohort of 111 nonagenarian women with a known pregnancy history using the well-characterised marker C-reactive protein (CRP) as the indicator. The observed CRP concentrations of these nonagenarians were elevated compared to those of young healthy women (19–30 years of age, n = 25) (CRP median 0.85 vs. 1.97 mg/l, Mann-Whitney U-test, p = 0.032), thus indicating the presence of inflammaging. The data demonstrated that there is a significant positive correlation between the CRP concentration and the number of children born (Spearman's rho 0.260, p = 0.006). When the numbers of sons and daughters born were analysed separately, we found a significant positive correlation only between CRP and the number of sons born (Spearman's rho 0.308, p = 0.001); no association between CRP concentration and the number of daughters born was detected (Spearman's rho 0.117, p = 0.223; Fig. 1 and Supplementary Fig. 1 for distribution of number of progeny). These data were also confirmed by analyzing the association of the CRP concentration and the number of sons in an ordinal logistic regression model with a *logit* link function (adjusted with the number of daughters). This model confirmed that only the number of sons was associated with the concentration of CRP (number of sons, proportional odds ratio (POR) = 1.77, p = 0.004; number of daughters POR = 1.32, p = 0.117).

**Figure 2:**
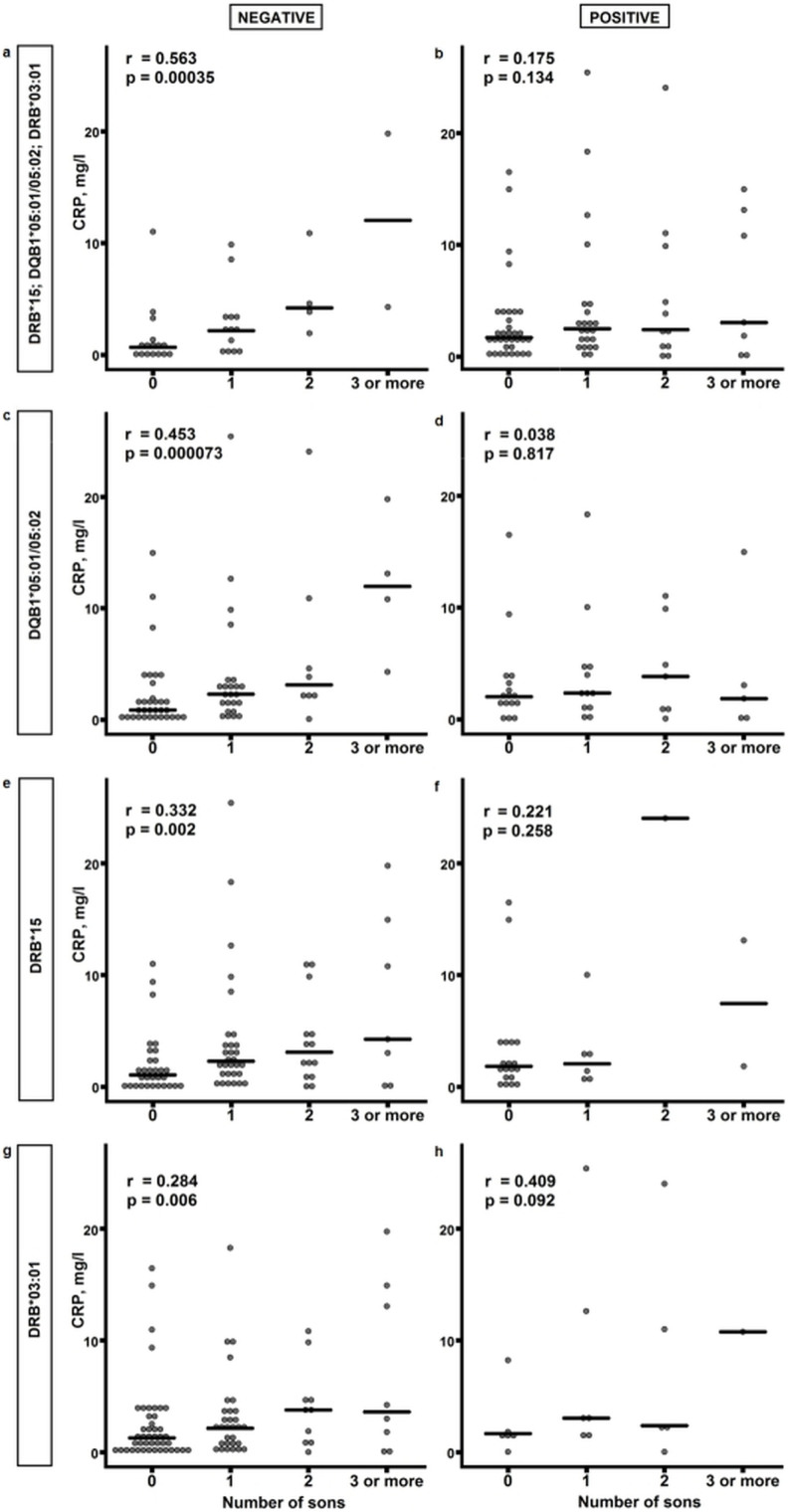
CRP concentration and HLA-genotype. CRP concentration in nonagenarian women grouped according to the number of sons and HLA genotype. (a) Individuals negative with respect to all HY-restricting alleles; (b) individuals positive with respect to one or more HY-restricting allele. (c) Through (h) individuals negative (left) and positive (right) with respect to the indicated single HY-restricting allele. Each dot represents one individual, and the horizontal lines correspond to the median of each group; r denotes Spearman's rho.

As the immune response against the Y-chromosomal transplantation antigen (HY), would be a likely candidate to explain this observation, we analysed the presence of three HLA class II alleles (DRB*15, DQB1*05:01/05:02, DRB3*03:01) known to be permissive restriction alleles enabling the anti-HY T cell response to occur^9^. In the absence of these alleles, a strong correlation between the CRP concentration and number of sons was evident (Spearman's rho 0.563, p = 0.00035); however, this association was not observed in individuals who possess one or more of these alleles (Spearman's rho 0.175, p = 0.134) (Fig. 2,). Furthermore, the results remained the same when the effect of these alleles were analysed separately (Fig. 2, Supplementary Table 1).

## Discussion

In summary, here we show that increased number of sons is associated with higher CRP concentration in elderly women, implying a potentiating effect of number of sons on inflammaging. This observation is in line with the theory of a trade-off between the energy invested in reproduction and somatic maintenance^4^,^5^ and also with the effect of the number of sons on longevity^6^,^7^. The adverse effect of sons born on maternal longevity has been proposed to be due to the higher energy consumption of a male pregnancy or social factors^6^,^7^. However, our results show that an association between number of sons and a biological marker can be observed up to 70 years after the male pregnancy, thus suggesting that there is also a biological component explaining the adverse effects of sons on maternal longevity.

The analysis of HLA alleles indicated that the association was restricted to individuals not carrying the alleles required for the anti-HY immune response to occur. Thus, it could be hypothesised that an effective anti-HY response is able to destroy those cells entering the mother during pregnancy or parturition, whereas in its absence, the number of transmitted cells is larger, and consequently the immune/inflammatory (graft vs. host and host vs. graft) responses are stronger. It could also be speculated that the observed mechanism is connected to fetal microchimerism (fMC). Fetal cells entering the mother during pregnancy are able to proliferate and differentiate in the maternal body, leading to a permanent fMC. In various studies fMC has been associated with pathogenesis of autoimmune diseases, such as systemic lupus erythematosus (SLE) and Sjögren's syndrome (SS) and a role in tumor formation or progression has also been suggested for fMC. Contradicting results have also been presented, particularly in cancer, where fetal cells have been proposed to have tissue repairing functions^10^,^11^. Nevertheless, our data suggest that the number of sons and HLA genotype should be taken in consideration in the further analysis of these disease associations.

These data have some limitations. It is very likely that inflammaging is not a uniform concept^12^. Thus, we do not know whether male cell-induced inflammation is similar to that induced/associated with, for example, degenerative processes. The concentration of CRP is only one component of the inflammaging phenotype, and it is affected by various biological variables, including aging-related pathologies. Moreover, an analysis of the clinical associations of the increased level of inflammaging associated with the number of sons born would require larger and more thoroughly characterised cohorts. However, taken together, these data show that the number of sons is one factor contributing to the increased systemic inflammation in nonagenarian women lacking the HY-restricting HLA alleles.

## Methods

### Study population

All study participants provided informed consent. The study was conducted according to the principles expressed in the declaration of Helsinki, and the study protocol was approved by the ethics committee of the city of Tampere (1592/403/1996).

The study population consisted of 111 nonagenarian women participating in the Vitality 90+ study, which is an ongoing prospective study involving individuals aged 90 years and older living in the city of Tampere, Finland. The individuals in this study were born in 1920 and were recruited as described previously^13^. The number of children born was collected from a questionnaire. The young controls (n = 25, aged 19 to 30 years, median 23 years) consisted of healthy laboratory personnel who were non-smokers, had no diagnosed chronic illnesses and had not been infected or had received vaccination in two weeks prior to the blood sample collection.

### CRP measurement from plasma

The blood samples were collected into EDTA-containing tubes by a trained medical student during a home visit. Plasma was separated by centrifugation for 15 min at 400 × g followed by transfer to new tubes and centrifugation for 15 min at 1000 × g. The plasma was stored at −70°C. The plasma CRP concentration was measured using Human CRP Immunoassay (Quantikine ELISA, R&D Systems, Minneapolis, MN, USA).

### HLA genotyping

Genotyping for HLA-DR/DQ alleles was based on sequence-specific hybridization using lanthanide-labeled oligonucleotide probes, as described earlier^14^. HLA-DRB1*15 was deduced from the presence of DQB1*06:02 and DRB1*05:02, as discerned by sequencing the hypervariable region of the second exon.

### Statistics and figures

The difference in the median concentration of CRP in plasma between nonagenarians and young controls was analysed using the Mann-Whitney U-test. The correlations between the number of progeny and the concentration of CRP were analysed using Spearman's rho. An ordinal logistic regression model adjusted with the number of daughters and with *logit* link function was used to confirm the association between the number of sons born and the concentration of CRP. The analyses were performed using IBM SPSS Statistics version 22 (IBM Corp., Sommers, NY, USA). The figures in the main manuscript were generated using R software. One individual with one son and a CRP concentration of 81.01 mg/l was omitted from the figures due to visualization reasons. This individual however was included in all the statistical calculations.

## Author Contributions

S.M. participated in the sample collection, data analysis and writing of the manuscript. T.N. participated in the data analysis and writing the manuscript. L.K. participated in the data analysis and writing of the manuscript. J.J. participated in the sample collection and data analysis. A.H. and M.J. were responsible for recruiting the study population. J.I. was responsible for H.L.A. genotyping. M.H. provided reagents and materials for the study and participated in the writing of the manuscript. All authors read and approved the final manuscript.

## Supplementary Material

Supplementary Information Supplementary info
