# Supplementary material for: Number of sons contributes to ageing-associated inflammation
Source: Sci Rep. 2015 Feb 27;5:8631. doi: 10.1038/srep08631 (PMC4342565; doi:10.1038/srep08631)
Supplement: Supplementary Information — Supplementary info [file srep08631-s1.pdf]

Number of sons contributes to ageing-associated inflammation

Saara Marttila[1,2]*, Tapio Nevalainen[1,2]*, Laura Kananen[1,2], Juulia Jylhävä[1,2], Marja Jylhä[2,3], Antti Hervonen[2,3], Jorma Ilonen[4,5], Mikko Hurme[1,2,6].

* These authors contributed equally to this work

[1]Department of Microbiology and Immunology, School of Medicine, University of Tampere, Tampere, Finland

[2]Gerontology Research Center, Tampere, Finland

[3]School of Health Sciences, University of Tampere, Tampere, Finland

[4]Immunogenetics Laboratory, University of Turku, Turku

[5]Department of Clinical Microbiology, University of Eastern Finland, Kuopio, Finland

[6]Fimlab Laboratories, Tampere, Finland

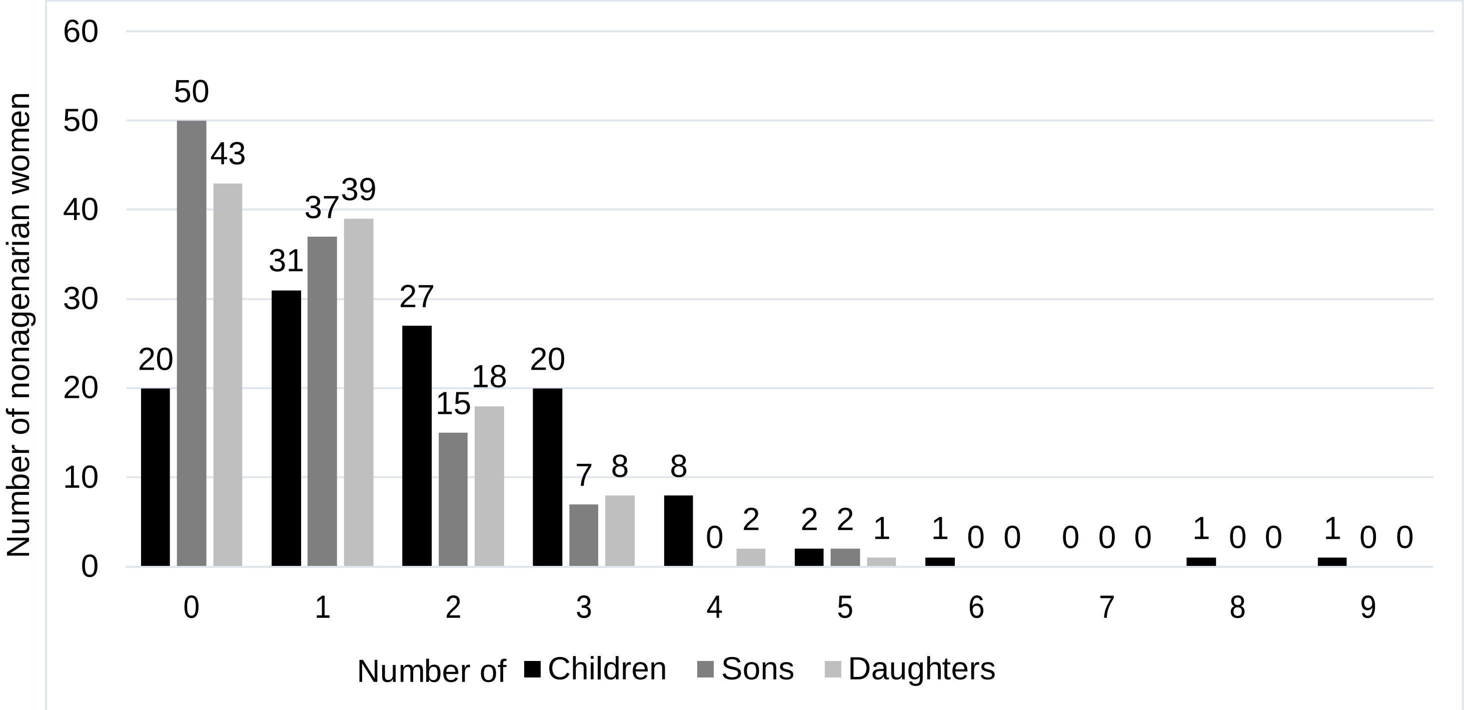

Supplementary figure 1. Distribution of the number of progeny in the study population. Each column represents the number of nonagenarian women with the given number of progeny.

	Number of sons -CRP		Number of daughters - CRP	
	Spearman's rho	p	Spearman's rho	p
All nonagenarians (n=111)	**0.308**	**0.001**	0.026	0.788
DRB*15 and DQB1*05:01/05:02 and DRB*03:01 negative (n=36)	**0.563**	**0.00035**	0.003	0.984
DRB*15 or DQB1*05:01/05:02 or DRB*03:01 positive (n=75)	0.175	0.134	0.199	0.087
DRB*15 negative (n=83)	**0.332**	**0.002**	0.091	0.411
DRB*15 positive (n=28)	0.221	0.258	0.201	0.304
DQB1*05:01/05:02 negative (n=71)	**0.453**	**0.000073**	0.024	0.845
DQB1*05:01/05:02 positive (n=40)	0.038	0.817	0.303	0.058
DRB*03:01 negative (n=93)	**0.284**	**0.006**	0.162	0.120
DRB*03:01 positive (n=18)	0.409	0.092	0.014	0.955

Supplementary table 1. Correlation of the CRP concentration and the number of sons or daughters in different genotype groups. The concentration of CRP is associated with the number of sons born when the entire study population is considered; however, the effect is restricted to individuals who do not have HY-restricting HLA alleles. This association is also observed when each HY-restricting allele is considered separately, even though there are individuals positive for one HY-restricting allele in a group negative with respect to another HY-restricting allele. No association between the number of daughters born and the level of CRP was identified. In terms of HY-restricting alleles, none of the study individuals were positive for all three, 11 were positive for two, and 64 were positive for one HY-restricting allele.
